# “Awake” ECCO_2_R superseded intubation in a near-fatal asthma attack

**DOI:** 10.1186/s40560-017-0247-7

**Published:** 2017-08-08

**Authors:** Thomas-Michael Schneider, Tibor Bence, Franz Brettner

**Affiliations:** Department of intensive care medicine, Krankenhaus Barmherzige Brueder Munich, Muenchen, Germany

**Keywords:** ECCO_2_R, Near-fatal asthma, ECMO

## Abstract

**Background:**

Near-fatal asthma attacks are life threatening events that often require mechanical ventilation. Extracorporeal carbon dioxide removal (ECCO_2_R) is, beside extracorporeal membrane oxygenation (ECMO), a well-established rescue option whenever ventilation gets to its limits. But there seems to be very rare experience with those techniques in avoiding mechanical ventilation in severe asthma attacks.

**Case presentation:**

A 67-year-old man with a near-fatal asthma attack deteriorated under non-invasive ventilation conditions. Beside pharmacological treatment, the intensivists decided to use an extracorporeal carbon dioxide removal system (ECCO_2_R) to avoid sedation and intubation. Within only a few hours, there was a breakthrough and the patient’s status improved continuously. One and a half days later, weaning from ECCO_2_R was already completed.

**Conclusions:**

The discussion deals with several advantages of extracorporeal lung support in acute asthma, the potential of avoiding intubation and sedation, as well as the benefits of a conscious and spontaneously breathing patient. Extracorporeal membrane oxygenation (ECMO) in general and ECCO_2_R in particular is a highly effective method for the treatment of an acute near-fatal asthma attack. Pathophysiological aspects favor the “awake” approach, without sedation, intubation, and mechanical ventilation. Therefore, experienced clinicians might consider “awake” ECCO_2_R in similar cases.

## Background

Extracorporeal CO_2_ removal (ECCO_2_R) is an extracorporeal membrane oxygenation (ECMO) subtype that is well established in intensive care medicine. Due to its characteristics, of mainly decarboxylation and to a lesser extent oxygenation, the major indication is hypercapnic lung failure as in chronic obstructive pulmonary disease (COPD). But it can also be used to facilitate mechanical ventilation in order to allow a protective ventilation regimen in other situations. Moreover, intubation and mechanical ventilation can be utterly avoided by using ECCO_2_R, making deep sedation or paralysis needless. With that approach, termed “awake”, the aim is to keep the patient interactive, without sedation and without mechanical ventilation support during ECCO_2_R. To the best of our knowledge, this is the first case report of managing a near-fatal asthma with ECCO_2_R in such an “awake” setting.

## Case presentation

A 67-year-old man with a history of intrinsic asthma, but who otherwise had very good health condition, was admitted to our intensive care unit with an acute asthma attack. Peripheral oxygenation saturation had been 80–89% without oxygen, and he then received supplemental oxygen, combined with inhalative salbutamol, as well as intravenous prednisolone, dimetindene, and ranitidine by an emergency physician at his home.

On admission to the intensive care unit, the patient was still awake, but severely tachy- and orthopnoic. Oxygen, 4 l min^−1^, resulted in 95% SpO_2_, but blood gas analysis showed a respiratory acidosis (pH 7.29, pCO_2_ 55 mmHg, pO_2_ 84 mmHg, HCO^3−^26.4, BE −1.6). Chest X-ray was normal, but severe expiratory wheezing could be heard on both lungs. The patient was on theophylline and formoterole/budesonide. Other relevant comorbidities were diabetes type 2 treated with metformin and coronary heart disease (1-vessel disease). He repeatedly inhaled salbutamol, ipatropiumbromide, and budesonide and received intravenous prednisolone, reproterole (bolus plus continuous), and magnesium. The patient was directly put on non-invasive ventilation (NIV; EVITA-4, Draeger®), facilitated with a cumulative dose of 4 mg morphine. While at first sight, the therapeutic regimen seemed to work and an ongoing deterioration with increasing pCO_2_-levels and signs of respiratory exhaustion were recognized. The patient’s vigilance became more and more impaired, and respiratory acidosis was later accompanied by a slight metabolic acidosis (blood gas analysis before ECCO_2_R: pH 7.24, pCO_2_ 61 mmHg, pO_2_ 289 mmHg, HCO^3−^26.1, BE −3.0). Meanwhile, other possible contributing causes, like pneumonia, lung embolism, and cardiac attack, were ruled out.

After careful consideration, and with the consent of the patient and his family, a ECCO_2_R system was prepared. Under non-invasive ventilation conditions and local anesthesia, a 22 French double lumen cannula (Twinport®, Novalung, Heilbronn, Germany) was placed in the right upper jugular vein under sonographic guidance. The system was started with a blood flow of 1 l min^−1^ and a sweep gas flow of 1 l min^−1^ oxygen, (ILA-activve®, Xenios, Heilbronn, Germany). Due to the respiratory effort of the patient along with hypovolemia, blood flow initially fluctuated between 0.6 and 1.5 l min^−1^. Fluid repletion was therefore conducted with balanced crystalloids combined with albumin to achieve a better intravascular effect. With the circuit running, a rapid improvement in almost all former deranged qualities was noticed: breath rate decreased from 40 to 16 per minute, SpO_2_ rose to 100%, and pCO_2_ was intentionally lowered very slowly (with 2 l min^−1^ sweep gas flow). Due to the sudden relief, the patient fell asleep for a few hours and NIV was down-graded to nasal oxygen (4 l min^−1^). Three hours after starting the ECCO_2_R circuit, blood gas analysis was normal (pH 7.39, pCO_2_ 44 mmHg, pO_2_ 93 mmHg, SpO_2_ 97%, BE 1.2). On day two, the patient was put on oral prednisolone (50 mg day^−1^). There was no more dyspnea or wheezing and both, nasal oxygen and sweep gas flow, could be reduced. Early mobilization and physiotherapy was started the very same day. Thirty-four hours after initiating the ECCO_2_R system, the patient was completely weaned, and the cannula could be removed without any complication. On day 4, the patient could be discharged from the ICU without need for supplemental oxygen and 6 days later, he left the hospital without any impairment.

## Discussion

Already having a high prevalence today, the Global Initiative for Asthma assumes that 400 million people will suffer from that disease by 2025 [1]. Near-fatal asthma is the most severe clinical presentation of asthma, with a high mortality if invasive ventilation is needed [2], which is the case in up to 30% of cases [3]. So far, there is no evidence for NIV being superior to invasive ventilation [4]. Altered sensorium, progressive exhaustion and ongoing respiratory acidosis are indicators for endotracheal intubation and ventilation [5]. The latter is known to cause adverse effects, like further lung injury, immobilization, and need for sedation and paralysis, with negative consequences for feeding, muscle activity, and delirium. In the case of an asthma attack, it is even able to exacerbate the pathophysiology: First, sedation and intubation are triggers of bronchospasms themselves [5]. Secondly, positive pressure ventilation in bronchial obstruction aggravates pre-existing hyperinflation, which results in further reduction of functional residual capacity and elevated intrathoracic pressures, thus affecting gas exchange and hemodynamic stability. In detail, it leads to ventilation-perfusion mismatch, decreased chest and lung extension, diaphragm dysfunction, compromised cardiac preload and output, and impaired pulmonary lymphatic drainage. Optimizing those factors is thought to favor lung recovery [6]. Therefore, adding pressure by initiating mechanical ventilation seems to be counter intuitive. In contrast, ECMO seems to perfectly work around those difficult issues, allowing spontaneous breathing, while the amount of ventilation volume that the patient is not able to carry out by himself, is accomplished by the extracorporeal circuit. Increasing the extracorporeal support results in a direct reduction of the patients’ respiratory effort, while the airways are left untouched and spontaneous breathing, with all its benefits for the healing lungs, is maintained. The advantages of keeping the patient “awake” are reduction of delirium, reduction of feeding problems, and allowing social contacts with friends and family, as well as allowing sufficient physiotherapy to reduce myopathy and critical care illness [6]. Conveniently, this can all be reached by placing just one double-lumen cannula, as low-flow systems like ECCO_2_R are completely satisfying for removing CO_2_ and oxygenation can be supported by just raising the nasal oxygen flow. The strong rationale for using vv-ECMO in conscious, spontaneous breathing patients has recently been published [6], while feasibility and safety of ECCO_2_R have also been demonstrated elsewhere [7]. Of course, there are not just benefits to mention but true difficulties and threats to consider, calling for caution and careful decision making: Cannula insertion in a spontaneously breathing patient, for example, is extremely dangerous because air embolism can occur. It can be prevented by thorough manual compression of the puncture site and application of continuous positive airway pressure at the same time. Aside from that, hemorrhagic events are frequent in ECMO therapy in general. The probably most intimidating scenario is an unexpected cannula displacement, maybe even provoked by an interactive patient, resulting in major hemorrhage and lethal shock, as time to react is short. Minor bleeding complications are often associated with therapeutic anticoagulation of the circuit or coagulation disorders. They can be managed with compression and normalizing coagulability. Last, but not the least, patient discomfort, pain, and anxiety in the “awake” approach might be in such an extent that starting deep sedation and mechanical ventilation is inevitable, and all advantages are lost. A high level of expertise in extracorporeal life support, as recently defined, and outstanding caring for such patients is therefore crucial [8]. From the economic point of view, the “awake” approach of lung support is clearly personnel and time consuming, as caretaking is even more demanding, compared to immobilized and sedated patients. Probably the most important factor for being successful with “awake” ECMO in such a case is the coincidence of a mentally robust patient, and a very experienced ECMO-team, being able to place the cannula fast enough, under extremely difficult conditions with just local anesthesia and without sedation.

## Conclusion

Pathophysiological aspects favor “awake” ECCO_2_R for the treatment of severe asthma attacks, which otherwise needed mechanical ventilation. This approach is perfect to fill in the gap between the ventilation volume the patient is able to breathe despite the asthma attack, and the one that they would need to fulfill their demands. With no need for mechanical ventilation negative effects can be avoided. Patients on ECMO or ECCO_2_R, without sedation and mechanical ventilation, benefit from several advantages for the healing lungs, as well as for mobility, nutrition, and cognition. Yet, it is a very challenging intervention, demanding a highly experienced ECMO-team with respect to the patients’ safety.

## Abbreviations

COPD: Chronic obstructive pulmonary disease
ECCO2R: Extracorporeal carbon dioxide removal
ECMO: Extracorporeal membrane oxygenation
NIV: Non-invasive ventilation
SpO2: Peripheral oxygen saturation
vv-(ECMO): Veno-venous (ECMO)
